# A single‐centre analysis of a biosimilar switching programme for adalimumab in inflammatory bowel disease

**DOI:** 10.1002/bcp.70086

**Published:** 2025-04-29

**Authors:** Louise Rabbitt, Áine Keogh, Linda Duane, John Ferguson, Anna Hobbins, Brian E. McGuire, Patrick Gillespie, Laurence J. Egan

**Affiliations:** ^1^ Discipline of Pharmacology and Therapeutics, School of Medicine University of Galway Ireland; ^2^ Department of Gastroenterology Galway University Hospitals Galway Ireland; ^3^ School of Mathematical and Statistical Sciences University of Galway Ireland; ^4^ Research Ireland Centre for Medical Devices (CÚRAM, 13/RC/2073_P2) Ireland; ^5^ Health Economics & Policy Analysis Centre (HEPAC), Institute for Lifecourse & Society (ILAS) University of Galway Ireland; ^6^ School of Psychology & Centre for Pain Research University of Galway Ireland

**Keywords:** adalimumab, biosimilar, health economics, inflammatory bowel disease, nocebo

## Abstract

**Aims:**

Amgevita is a licensed biosimilar to adalimumab, having demonstrated high pharmacokinetic and clinical similarity to Humira. Switching to a lower‐cost medicine may elicit a nocebo effect, whereby expectations of poorer efficacy impact outcomes despite pharmacological similarity. This prospective cohort study examined clinical and economic outcomes and associated psychosocial variables in a group of patients undergoing a nonmedical switch to biosimilar adalimumab.

**Methods:**

Patients with inflammatory bowel disease (IBD) were followed before and after switching from Humira to Amgevita. Objective disease activity was assessed pre‐ and post‐switch using the Harvey–Bradshaw Index (Crohn's disease) or partial Mayo score (ulcerative colitis), faecal calprotectin and C‐reactive protein. Subjective symptom burden was measured using the IBD Control Questionnaire (IBDCQ). Pre‐switch, health anxiety was measured using the Health Anxiety Index (HAI).

**Results:**

In total, 64 patients aged 18–67 were enrolled. IBDCQ scores marginally improved post‐switch (13.33 *vs*, 12.49, *P =* .043), with no significant changes in objective disease activity scores, faecal calprotectin or C‐reactive protein. Sixteen patients reported 17 new adverse events within 4 weeks. Logistic regression revealed a significant relationship between HAI scores and adverse events (*P =* .0079); each unit increase in HAI score increased the odds of reporting an adverse event by 21%. Drug cost savings for the 64 patients over 8 weeks totalled €143 958.

**Conclusion:**

Switching to biosimilar adalimumab did not affect disease control or quality of life. 25% of patients developed new side effects, particularly those with high levels of health anxiety. Significant cost savings were achieved.

**What is already known about this subject**

- Biosimilar adalimumab has been proven a safe, effective and cost‐effective alternative to the reference product, Humira, in treating inflammatory bowel disease.
- Nonmedical switching from reference product to biosimilar is recommended in many jurisdictions to improve cost‐effectiveness of biologic therapy and potentially improve access to treatment for patients with inflammatory bowel disease

**What this study adds**

- This study illustrates the real‐world experience of switching to biosimilar adalimumab using a dedicated switching clinic in 1 centre.
- The cost‐saving and economic impact, including the cost of an extra switching clinic, is described.

## 1 INTRODUCTION

*Biologic* drugs are large‐molecule monoclonal antibodies against specific molecular targets. They have become commonplace in treatment of inflammatory bowel disease (IBD), with consequent significant cost implications.^1^ Similar in concept to a *generic* version of a traditional small molecule drug, biosimilar drugs are licensed when the original reference product (RP) is off patent. Due to the complex nature of these molecules and to variability in the production methods, minor differences exist between biosimilars and reference products. However, regulatory approval of a biosimilar medicine requires demonstration of a high degree of pharmacological similarity and lack of clinically significant differences in efficacy and safety in at least 1 of the clinical indications of the RP.

Amgevita is a licensed biosimilar of adalimumab, having demonstrated a high degree of similarity to the RP (Humira) in pharmacokinetic and clinical trials.^2^, ^3^ It is a citrate‐free formulation of adalimumab chosen in line with national pharmacoeconomic guidance.^4^ Successful nonmedical switches to biosimilar adalimumab were initially demonstrated in rheumatic joint disease, and have subsequently been demonstrated in IBD.^5^, ^6^ As a proportion of total health spending, Ireland spends more than the EU average on pharmaceuticals.^7^ Given the considerable cost saving possible by switching from reference products to biosimilars, the national Medicines Management Programme in Ireland recommends switching patients from Humira to a biosimilar such as Amgevita.^4^

While randomized studies have shown Amgevita to be equivalent to Humira, concerns persist among patients and prescribers as to its efficacy and interchangeability; this is reflected in a significant lag time between regulatory approval and clinical acceptance.^8^ Patients switching to a medicine they perceive as lower cost may experience a nocebo effect, whereby an expectation of poorer efficacy or more adverse effects may lead to worse clinical outcomes despite pharmacologically identical properties.^9^, ^10^, ^11^, ^12^ While higher rates of discontinuation in open‐label biosimilar trials compared with blinded trials suggest nocebo effects occur in this setting, limited data exist on the magnitude and timing of a nocebo effect to be expected with biosimilar switching.^13^ While expectation, verbal suggestion or mention of possible adverse effects, prior conditioning, psychological characteristics such as anxiety and situational factors have all been posited to contribute to nocebo effects,^14^, ^15^ relatively little is known about what patient factors may predict the development of nocebo effects in this setting.

The Irish healthcare system involves a complex mix of public and private finance and provision. Public finance accounts for 75% of total health expenditure, while out‐of‐pocket payments and voluntary health insurance accounts for 11.7 and 14%, respectively.^7^ Approximately 1/3 of the Irish population have Medical Cards, a means‐tested system that covers medical expenses such as doctor visits and prescribed medicines; such patients pay €1.50 per item at the point of dispensing.^16^ In addition, all citizens are eligible for the Drugs Payment Scheme, which caps medicines costs at €80 per household per month. In the case of self‐administered monoclonal antibodies such as adalimumab, prescriptions are written by hospital‐based providers and dispensed from community‐based pharmacies through the Primary Care Reimbursement Scheme.

We hypothesized that in patients who self‐administer biosimilar medicines, there will be a nocebo effect in some patients which may be predicted by certain variables (e.g. age, sex, health anxiety, beliefs about medicines, duration of diagnosis, educational attainment), which will result in an unmeasured health and economic burden. In this prospective cohort study, we studied a group of patients with IBD (Crohn's disease and ulcerative colitis) undergoing a nonmedical switch to biosimilar adalimumab to examine clinical outcomes, associated psychosocial variables and healthcare costs.

## 2 METHODS

In August 2020, all patients treated with adalimumab for IBD in our hospital (approximately 140 patients) were invited to switch to biosimilar adalimumab (Amgevita, manufactured by Amgen Ltd). An Advanced Nurse Practitioner (ANP) in IBD carried out a telephone‐based clinic during which patients were informed of the rationale for the switch, appropriate information given and their questions answered. They were then given a new prescription to use for their next dose of adalimumab. A proportion of these patients were, additionally, invited to participate in this research study. Prior to the switch to biosimilar, study participants were asked to fill out questionnaires administered using the online platform LimeSurvey. The remaining patients who did not enter the study underwent biosimilar switching with routine clinical follow‐up and no additional measures.

Two patient‐reported outcome measures were administered. Participants' subjective experience of their IBD symptoms was measured using the IBD Control Questionnaire (IBDCQ) 4 weeks pre‐switch and 4 weeks post‐switch. This is a well‐established and validated measure of patient‐experienced IBD control, which uses 8 questions scored from 0 to 16 where higher scores indicate lower symptom burden and better disease control, and a 0–100 visual analogue scale (VAS) to rate overall disease control where 100 indicates the best possible control.^17^, ^18^ Health‐related quality of life was measured using the EQ‐5D‐5L Utility score and VAS 8 weeks pre‐switch and again 8 weeks post‐switch. This measures quality of life over 5 domains (anxiety/depression, mobility, self‐care, usual activities and pain/discomfort) while the utility score applies scores referenced against Irish‐specific norms.^19^

Objective disease control was scored using objective disease scoring systems administered 4 weeks pre‐switch and 4 weeks post‐switch: the Harvey–Bradshaw Index for Crohn's disease and the partial Mayo score for ulcerative colitis,^20^, ^21^ and biochemical measures of inflammation including faecal calprotectin (FC) and serum C‐reactive protein (CRP). Duration of IBD diagnosis and duration of Humira treatment were also measured.

The Beliefs about Medicines Questionnaire (BMQ) was developed to assess cognitive representations of medicines and includes measures of how necessary a person considers their medicines to be (e.g. “Without Humira I would be very ill”) and concerns they have about taking their medicines (e.g. “Sometimes I worry about the long‐term effects of taking Humira”). The difference between these scores is referred to as the necessity‐concerns differential index and has been shown to predict medication adherence.^22^ The short‐form Health Anxiety Index (SHAI) is a widely used, valid and reliable measure of health anxiety.^23^ The SHAI requires participants to indicate their agreement with a series of 18 statements (e.g. “I spend most of my time worrying about my health”) and generates a score from 0 to 54. Patients with hypochondriasis and anxiety have demonstrated score norms of 37.9 and 18.5, respectively.^23^ The BMQ and SHAI were both measured 8 weeks pre‐switch.

A nocebo effect was defined as either: (i) a worsening in subjective scores of IBD‐related symptoms using the IBDCQ in the absence of objective evidence of an increase in disease activity measured using objective disease scoring systems and biochemical measures of inflammation; or (ii) the emergence of new side effects attributed to the biosimilar. An adverse event was defined as any untoward medical occurrence in a participant. During follow‐up telephone consultations, participants were asked about the occurrence of new side effects using open‐ended questions with neutral prompts by a staff nurse or ANP and their responses were recorded narratively.

A cost analysis was conducted that focused on the healthcare resources associated with implementing the biosimilar switching programme, drug costs and the use of unscheduled primary and secondary care over an 8‐week follow up period. To estimate the direct implementation costs of this switching clinic, a cost analysis considered the staff time required to plan the switching programme, identify patients for switching, create patient information literature and facilitate 12 telephone switching clinics over a 2‐month period, staffed by an ANP and staff nurse in IBD. Data on use of staff time for the switch were collected prospectively. Unit costs were identified from publicly available data sources including the Irish health service published salaries of clinician and clerical staff. Salary costs were estimated on the basis of the relevant Health Service Executive salary scales and following Irish health economic guidance.^24^, ^25^ Drug dose usage for Humira and Amgevita for an 8‐week period was costed using the Primary Care Reimbursement Scheme unit cost database. Unscheduled use of general practice, hospital consultant, nonconsultant doctor and nurse practitioner and outpatient clinic care were identified from available medical records for an 8‐week period, and costed using unit costs from published sources as mentioned earlier. Unit cost estimates, where necessary, were transformed to Euros (€) in 2021 prices using appropriate indices.^26^

The primary analysis compared subjective and objective symptom scores, 4 weeks prior to medication switch with the corresponding scores 4 weeks post‐switch, using 2‐sample paired *t*‐tests for outcomes with approximately normal distributions and the Wilcoxon signed rank test for other continuous outcomes. Factors associated with the presence of new adverse events post‐switch were investigated using logistic regression models, while factors associated with change in subjective symptom scores were investigated using multiple regression. In all cases, statistical significance was judged based on *P* < .05, and 95% confidence intervals (CIs) were reported with point estimates. A descriptive analysis of healthcare costs, in terms of total and mean per patient estimates, are presented.

Sample size was estimated using paired *t*‐test to determine the likely difference between reference product and biosimilar, with respect to the primary endpoint: symptoms as measured using the IBDCQ VAS at 4 weeks. Background variability in the data was estimated using IBDCQ VAS results from a cohort of IBD patients taking Inflectra (biosimilar Infliximab; a comparable population). A sample size of 47 participants was required to achieve a 5% significant *P*‐value (for a 1‐sided test for decrease in scores at 80% power) when the true decrease in means is 10%. To allow for dropouts, extra participants were recruited. In total, 71 participants joined this cohort study. Each patient provided written informed consent to participate. Ethical approval was granted by the Galway University Hospital Clinical Research Ethics Committee (Ref. C.A.2346).

Key protein targets and ligands in this article are hyperlinked to corresponding entries in http://www.guidetopharmacology.org, and are permanently archived in the Concise Guide to PHARMACOLOGY 2021/22.^27^

## 3 RESULTS

**FIGURE 1:**
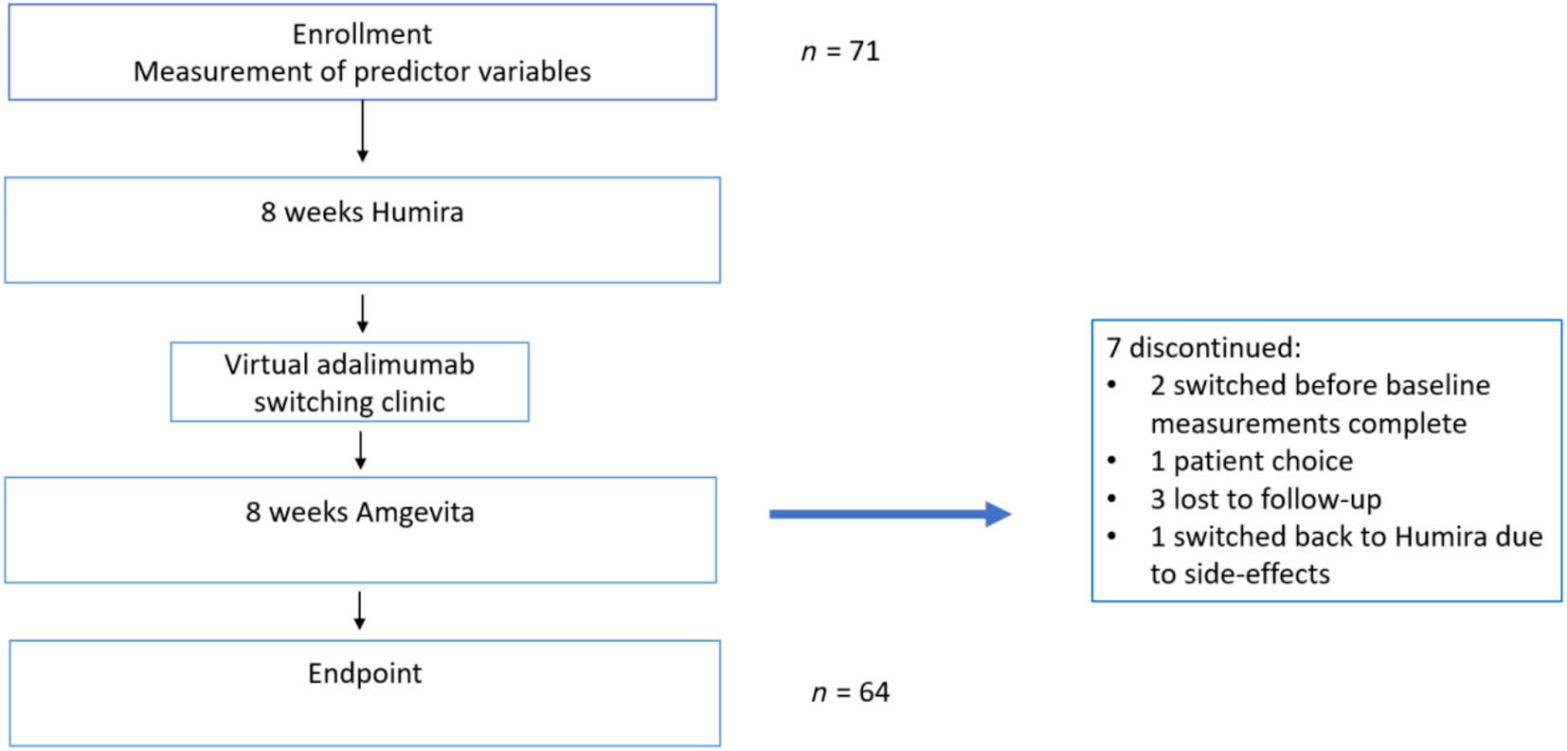
Flowchart.

Of the 71 patients initially recruited, 7 were excluded from this analysis: 2 switched before their baseline measurements were complete, 3 were lost to follow‐up, 2 left the study due to patient choice, of whom 1 switched back to Humira (Figure 1). The characteristics of the included cohort are shown in Table 1. Crohn's disease was the predominant diagnosis. Of the 60 patients who responded to the economic questionnaire, 38 (63%) held a Medical Card. On average, participants had a diagnosis of IBD for 12.38 years (standard deviation 8.07, range 2–31), and had been taking adalimumab for 3.66 years (standard deviation 3.48 range 0.13–15.16)​ prior to the switch.

**TABLE 1:** Participant characteristics.

*N*	64​
Female (%)	37 (58%)
Age (years; range)	41.79 (18.63–67.44)
Crohn’s disease (*n*)	50 (78%)
Ulcerative colitis (*n*)	11 (17%)
Indeterminate colitis (*n*)	3 (5%)
Hx previous surgery (*n*)	26 (40.6%)
Duration of diagnosis (years)	12.38 (2–31)
Duration of Humira treatment (years)	3.66 (0.13–15.16)

To minimize bias due to possible time trends and minimize missing data, the difference in symptom scores on the most recent visit before and after the switch was analysed (4 weeks pre‐switch and 4 weeks post‐switch). IBDCQ scores were marginally lower after the switch (from 13.33 4 weeks pre‐switch, to 12.49, 4 weeks post‐switch; *t* = −2.0714, df = 56, *P* = .04295). There was no significant different in health‐related quality of life as measured 8 weeks pre‐switch and 8 weeks post‐switch. Mean EQ‐5D‐5L utility score was 0.88 preswitch and 0.85 postswitch (*t* = −1.1306, df = 36, *P =* .27 [95% CI for difference: −0.11, 0.03]) and the mean EQ‐5D‐5L VAS was 79.67 preswitch and 78.90 post switch (*t* = 0.20463, df = 35, *P =* .84 [95% CI for difference: −6.20, 7.58]).

Before switching, no participants reported adverse effects with adalimumab treatment, as may be expected from a cohort who are well‐established on a drug. Sixty‐two participants provided follow‐up information about adverse effects; of these, 16 participants reported 17 new adverse effects after the biosimilar switch. Most of these (11/16) were reported within the first 4 weeks postswitch. There were 6 reports of injection site reactions including stinging and pain at injection site. Eleven patients reported other symptoms: 4 patients reported headache, 2 patients reported malaise, 2 reported joint pain, 2 reported nausea, 1 reported low back pain, 1 reported fatigue and 1 reported a skin rash (Supplemental Table S4). At 8‐weeks post‐switch follow‐up, no adverse effects were reported. Given possible differences between the auto‐injector devices between RP and biosimilar, we focussed on the 11 noninjection‐site adverse effects. McNemar's test showed 11 adverse effects post‐switch (with no adverse effects pre‐switch) to be significant (α = .05; *P =* 9.8 × 10^−4^).

Two patients reverted back to Humira during the study: 1 reported headache, nausea and presyncope, which started within 1 hour of taking Amgevita and lasted 5–7 days after both of 2 doses of Amgevita; another reported worsening of an existing skin rash, joint pain and joint stiffness after 2 doses. Both patients were seen for an additional out‐patient appointment and switched back to Humira at their request.

No significant difference was seen in disease activity measured using objective disease activity scores pre‐ and post‐switch. There was no significant change in mean Harvey–Bradshaw Index scores after the switch (2.8 pre‐switch *vs*. 2.1 post‐switch; t = −1.2989, df = 42, *P =* .2011; Figure S1). Given the small UC population (11 patients with recorded measurements), there was no substantial power comparison of mean partial Mayo score pre‐ and post‐switch, but there was no evidence of a true change post‐switch (0.3 pre‐switch and 0.6 post‐switch; *t* = 1.1744, df = 10, *P =* .2674).

Twenty‐four patients had paired FC results. The median FC was 79.5 μg/g pre‐switch and 61 μg/g post‐switch. The difference in FC before and after switching was not significant (Wilcoxon signed rank test with continuity correction V = 72, *P =* .8564; Figure S2). The difference between CRP pre‐switch and post‐switch was not significant (mean 2.3 pre‐switch and 2 post‐switch; *t* = −1.0476, df = 40, *P =* .3011; Figure S3). There was no evidence of a change in biochemical markers of disease activity pre‐ and post‐switch. Opportunistic adalimumab levels were checked in 44 patients, of whom 39 had a level within the therapeutic range, and 2 had anti‐adalimumab antibodies present.

Individual marginal logistic regressions with a single explanatory variable showed no significant correlation between the experience of new adverse effects and age, sex, diagnosis, duration of diagnosis, duration of Humira, educational attainment or the BMQ necessity‐concerns index. However, there was a significant relationship with SHAI (*P =* .0079). Each 1 unit increase in the SHAI score increased the odds of having an adverse event by about 21% (Table 2). There was no significant correlation between IBDCQ scores and age, sex, diagnosis, duration of diagnosis, duration of Humira, educational attainment, SHAI (Figure S4), or BMQ scores (including the necessity‐concerns index; Table 3).

**TABLE 2:** Prediction of new adverse effects.

	*P* value
Age	.72
Sex	.31
Diagnosis	.12
Duration of diagnosis	.48
Duration of Humira	.35
Educational attainment	.43
HAI	.0079*
BMQ Specific Necessity subscore	.51
BMQ Specific Concerns subscore	.91
BMQ Necessity–Concerns index	.68

Abbreviations: BMQ, Beliefs about Medicines Questionnaire; HAI, Health Anxiety Index.

**TABLE 3:** Prediction of change in inflammatory bowel disease Control Questionnaire.

	*P* value​
Age	.52
Sex	.96
Diagnosis	.17
Duration of diagnosis	.49
Duration of Humira	.22
Educational attainment	0.55
HAI	0.72
BMQ Specific Necessity subscore	0.92
BMQ Specific Concerns subscore	0.49
BMQ Necessity–Concerns Index	0.5288

Abbreviations: BMQ, Beliefs about Medicines Questionnaire; HAI, Health Anxiety Index.

### 3.1 Healthcare costs

The cost per patient of the switching clinic was calculated based on the initial 71 patients recruited. The total staff cost of running switching clinics for 71 patients was €9464, giving an average cost of €133 per patient (Table S1). An incentivization programme was in operation at the time of this switching programme, which provided a gainshare incentive of €500 per patient switched which was paid to the local hospital department. This resulted in a €32 000 once‐off payment to our department for switching 64 patients. This payment was not included in the cost analysis.

In addition to the scheduled switching clinic, the switch in this cohort was followed by unscheduled care needs in 4/64 (6%) patients within the 8 weeks of follow‐up. Two of these were patients who experienced adverse effects requiring extra telephone calls, GP and outpatient visits and in 1 case, an ambulance transfer to hospital. One patient was incidentally noted to have a subtherapeutic adalimumab level and positive anti‐adalimumab antibodies requiring an extra outpatient review pre‐switch. One patient was admitted to hospital with symptoms of a flare requiring endoscopic examination and 2‐night inpatient admission; these symptoms preceded the switch. Because the latter 2 of these were deemed unrelated to the switch (because they occurred before switching), the cost of their unscheduled care is not considered in this analysis. For the 2 patients with unscheduled care needs related to the switch, the total cost of unscheduled care was €1622 (Table S2), giving a mean cost per‐patient of unscheduled care over an 8‐week time horizon of €25.

Of the 64 patients in this analysis, 44 were prescribed Humira 40 mg every 2 weeks, 19 were prescribed Humira 40 mg weekly and 1 was prescribed 80 mg every 2 weeks. Considering the differing costs of different doses, the mean cost of Humira per patient per month in this cohort was €2865, compared with €1740 for Amgevita, giving a mean cost saving of €1125 per patient per month in drug costs^28^ (Table S3). The total drug cost saving for these 64 patients can be estimated at €864 000 per year. Within the 8‐week time horizon for this analysis, the total drug cost saving for these 64 patients was €143 958; taking into account the switching clinic cost and the cost of unscheduled care post‐switch, the cost saving per patient was €2091 (Table 4).

**TABLE 4:** Cost comparison, 8‐week time horizon. *N* = 64.

	Humira (€)	Amgevita (€)
Mean total drug cost per patient	5729.19	3479.86
Switching clinic cost per patient	0.00	133.00
Mean unscheduled care cost per patient	0.00	25.35
Total cost per patient (2 months)	5729.19	3638.20
Cost saving per patient (2 months)		2090.98

## 4 DISCUSSION

In this prospective cohort study, we describe the experience of a nonmedical biosimilar adalimumab switching programme in a single gastroenterology unit, with a particular focus in this study on predicting nocebo effects.

There is some evidence of mild improvement of subjective symptoms (IBDCQ score) after switching to the biosimilar, though the actual difference in score was so small that it may be of questionable clinical significance. There was no evidence that switching affected objective disease scores or biochemical parameters (FC, CRP); this supports the therapeutic equivalence of originator adalimumab and biosimilar adalimumab and is consistent with other studies of nonmedical biosimilar switching.^5^, ^29^, ^30^, ^31^ Rates of reported adverse events seemed to increase after the switch. There was some evidence that reported adverse events (post‐switch) depended on the health anxiety score. A similar analysis showed no evidence that the health anxiety score was related to change in IBDCQ score. No evidence of other relationships between variables, including disease duration and duration of adalimumab treatment, and reported side effects or reported symptoms was found.

The rate of adverse events in this cohort is significantly higher than rates reported in the international literature.^32^ This could be the result of how such events were defined, the unstructured way they were recorded, or the timing of when patients were asked to report. Any untoward medical occurrence in a participant, based on participants self‐report, was included as an adverse effect, regardless of severity, and there was no formal assessment of causality. Future research could apply a more rigorous framework to further elucidate the relationship between biosimilar switching and the occurrence of new adverse effects. In this study, 2 patients reverted to Humira as a result of adverse effects attributed to biosimilar adalimumab. Nocebo effects have been posited as a reason for discontinuation in several observational studies but given the lack of randomized data, cannot be confirmed to affect medication persistence.^33^

The pragmatic design and real‐world setting provide good ecological validity, and the collection of prospective longitudinal clinical data lend the study design strength. As this is an observational study with no blinded comparator group, the adverse effects described in this cohort cannot be conclusively attributed to nocebo effects. The frequent reports of injection site reactions may reflect differences in the design and function of the auto‐injector appliances between the RP and biosimilar. Moreover, there may potentially be a nonresponder bias given the high rate of nonresponders to follow‐up questionnaires which may have been associated with the study taking place during the Covid‐19 pandemic. Endoscopic clinical data are not included in this study due to the short follow‐up period and resource constraints. The open‐label design, and lack of a control group are further limitations of this study. We also note the lack of a measure to capture participants' expectations before the switch took place.

Expediting switching through dedicated switching clinics, while time consuming, has the potential for significant cost advantages. It is acknowledged that this study represents an estimation of the direct implementation costs only and does not take into consideration a range of direct and indirect costs within and outside of the healthcare system. Additional infrastructural requirements such as an office space were not factored into the analysis based on the assumption that these clinics could be performed within the existing physical infrastructure. However, that assumption is contingent on services being reorganized to facilitate introduction of switching clinics. In the event that such space would not be available, it may be necessary to consider the additional overhead costs and potential disruption to other services a clinic such as this may cause. Nonetheless, the considerable monthly drug cost savings, balanced against the cost of running a dedicated switching clinic, demonstrates significant benefit to taking a proactive approach to switching instead of opportunistic switching at annual or semiannual out‐patient review visits. The majority of the cost in the switching clinic was due to planning and setup, meaning that for greater numbers of patients the cost per patient would be lower, further improving the cost‐efficiency of this intervention. Incentivization schemes, of the type described here, may also be cost‐effective if they expedite a biosimilar switch. Moreover, a proactive approach such as this allows for biosimilar switching to take place with appropriate patient educational resources and well‐informed staff, which may help to address the logistical challenges of switching and minimize potential nocebo effects.^34^ Regardless of the exact mechanism, a nonmedical switch should be based on collaborative, patient‐focused decision‐making.^33^ An economic evaluation would be required to definitively assess the cost effectiveness of such programmes.

This study adds to the growing literature describing the real‐life experience of using biosimilar medicines and will be relevant to clinicians planning nonmedical biosimilar switches in diverse settings. There is a need for ongoing education and support for prescribers using biosimilar medicines.^35^ Healthcare professionals confident in their knowledge of biosimilars and aware of bias‐inducing factors may help reduce the risk of nocebo effects and improve patients' outcomes.^9^, ^36^

Future research should aim to further elucidate whether nocebo effects can be minimized or prevented, perhaps through the delivery of balanced information on risk–benefit profiles, framing information to focus on positive attributes, and promoting shared decision‐making processes along with patient empowerment. The relationship between health anxiety and the occurrence of adverse effects in this study may suggest that health anxiety could be an appropriate target for intervention to improve outcomes, or that prescribers should offer additional assistance to those with high levels of health anxiety at the time of biosimilar switching.^37^ Future longer‐term studies are necessary to confirm safety, efficacy and abeyance of adverse effects after switching.

## 5 CONCLUSIONS

In this single‐centre prospective cohort study of patients with IBD, switching to biosimilar adalimumab was found to be safe and effective within the short follow‐up period, in line with international experience of this and other biosimilar medicines.^38^, ^39^, ^40^ With biosimilar switching, prescribers may expect some patients to develop new side effects, particularly those with high levels of health anxiety.

## AUTHOR CONTRIBUTIONS

L.R., A.K., L.J.E, B. M and P. G conceived and designed the study. A.K. and L.D. completed the switching clinics and the distribution and collection of questionnaire responses. P.G. and A.H. supervised and directed the economic analysis. J.F. designed and performed the statistical analysis. L.R. drafted the manuscript. All authors helped critically revise the manuscript and approved the final version for publication.

## CONFLICT OF INTEREST STATEMENT

The authors declare that they have no conflicts of interest related to the content of this manuscript.

## Supporting information


**FIGURE S1** Harvey–Bradshaw Index (HBI) scores pre‐ and postswitch.
**FIGURE S2** Faecal calprotectin (FC; measured in μg/g) measurements pre‐ and postswitch.
**FIGURE S3** C‐reactive protein (CRP) measurements pre‐ and postswitch.
**TABLE S1** Switching clinic staff costs.
**TABLE S2** Unscheduled care costs (2 patients, 8‐week time horizon).
**TABLE S3** Mean drug cost calculation.
**TABLE S4** Occurrence of adverse effects.

## Data Availability

The data that support the findings of this study are available on request from the corresponding author. The data are not publicly available due to privacy or ethical restrictions.

## References

[1] Van Der Valk ME , Mangen MJJ , Leenders M , et al. Healthcare costs of inflammatory bowel disease have shifted from hospitalisation and surgery towards anti‐TNFα therapy: results from the COIN study. Gut. 2014;63(1):72‐79. doi:10.1136/gutjnl-2012-303376 23135759

[2] Papp K , Bachelez H , Costanzo A , et al. Clinical similarity of biosimilar ABP 501 to adalimumab in the treatment of patients with moderate to severe plaque psoriasis: a randomized, double‐blind, multicenter, phase III study. J am Acad Dermatol. 2017;76(6):1093‐1102. doi:10.1016/j.jaad.2016.12.014 28291552

[3] Cohen S , Genovese MC , Choy E , et al. Efficacy and safety of the biosimilar ABP 501 compared with adalimumab in patients with moderate to severe rheumatoid arthritis: a randomised, double‐blind, phase III equivalence study. Ann Rheum Dis. 2017;76(10):1679‐1687. doi:10.1136/annrheumdis-2016-210459 28584187 PMC5629940

[4] “Medicines Management Programme.” Best‐value biological medicines: Adalimumab & Etanercept. https://www.hse.ie/eng/about/who/cspd/medicines-management/best-value-medicines/best-value-biological-medicines/

[5] Casanova MJ , Nantes Ó , Varela P , et al. Real‐world outcomes of switching from adalimumab originator to adalimumab biosimilar in patients with inflammatory bowel disease: the ADA‐SWITCH study. Aliment Pharmacol Ther. 2023;58(1):60‐70. doi:10.1111/apt.17525 37089065

[6] van Adrichem RCS , Voorneveld HJE , Waverijn GJ , Kok MR , Bisoendial RJ . The non‐medical switch from reference adalimumab to biosimilar adalimumab is highly successful in a large cohort of patients with stable inflammatory rheumatic joint diseases: a real‐life observational study. Rheumatol Ther. 2022;9(4):1109‐1118. doi:10.1007/s40744-022-00465-6 35655028 PMC9314483

[7] OECD . State of health in the EU. Country Health Profile; 2021. Published online 2021:1–23. https://ec.europa.eu/health/sites/health/files/state/docs/2019_chp_be_english.pdf

[8] Duggan B , Smith A , Barry M . Uptake of biosimilars for TNF‐α inhibitors adalimumab and etanercept following the best‐value biological medicine initiative in Ireland. Int J Clin Pharmacol. 2021;43(5):1251‐1256. doi:10.1007/s11096-021-01243-0 33560486

[9] Colloca L , Panaccione R , Murphy TK . The clinical implications of nocebo effects for biosimilar therapy. Front Pharmacol. 2019;10(November):1‐11. doi:10.3389/fphar.2019.01372 31849647 PMC6895996

[10] Colloca L , Miller FG . The nocebo effect and its relevance for clinical practice. Psychosom Med. 2011;73(7):598‐603. doi:10.1097/PSY.0b013e3182294a50 21862825 PMC3167012

[11] Faasse K , Cundy T , Gamble G , Petrie KJ . The effect of an apparent change to a branded or generic medication on drug effectiveness and side effects. Psychosom Med. 2013;75(1):90‐96. doi:10.1097/PSY.0b013e3182738826 23115341

[12] Espay AJ , Norris MM , Eliassen JC , et al. Placebo effect of medication cost in Parkinson disease. Neurology. 2015;84(8):794‐802. doi:10.1212/WNL.0000000000001282 25632091 PMC4345649

[13] Odinet JS , Day CE , Cruz JL , Heindel GA . The biosimilar nocebo effect? A systematic review of double‐blinded versus open‐label studies. J Manag Care Spec Pharm. 2018;24(10):952‐959. doi:10.18553/jmcp.2018.24.10.952 30247100 PMC10398229

[14] Webster RK , Weinman J , Rubin GJ . A systematic review of factors that contribute to nocebo effects. Health Psychol. 2016;35(12):1334‐1355. doi:10.1037/hea0000416.supp 27657801

[15] Barsky AJ , Saintfort R , Rogers MP , Borus JF . Nonspecific medication side effects and the nocebo phenomenon. Sci Eng Ethics. 2004;10(1):133‐134. 10.1001/jama.287.5.622 11829702

[16] “ Central Sstatistics Office .” Coverage of Essential Health Services. https://www.cso.ie/en/releasesandpublications/ep/p-sdg3/irelandsunsdgs2019-reportonindicatorsforgoal3goodhealthandwell-being/healthcare/

[17] Bodger K , Ormerod C , Shackcloth D , Harrison M . Development and validation of a rapid, generic measure of disease control from the patient's perspective: the IBD‐control questionnaire. Gut. 2014;63(7):1092‐1102. doi:10.1136/gutjnl-2013-305600 24107590 PMC4078750

[18] Gebeyehu GG , Taylor F , Dobson L , et al. Validation of the IBD‐control questionnaire across different sociodemographic and clinical subgroups: secondary analysis of a nationwide electronic survey. J Crohns Colitis. 2024;18(2):275‐285. doi:10.1093/ecco-jcc/jjad147 37706542 PMC10896631

[19] Hobbins A , Barry L , Kelleher D , et al. Utility values for health states in Ireland: a value set for the EQ‐5D‐5L. Pharmacoeconomics. 2018;36(11):1345‐1353. doi:10.1007/s40273-018-0690-x 30051267 PMC6182460

[20] Lewis JD , Chuai S , Nessel L , Lichtenstein GR , Aberra FN , Ellenberg JH . Use of the noninvasive components of the Mayo score to assess clinical response in ulcerative colitis. Inflamm Bowel Dis. 2008;14(12):1660‐1666. doi:10.1002/ibd.20520 18623174 PMC2597552

[21] Harvey RF , Bradshaw JM . A simple index of Crohn's‐disease activity. Lancet. 1980;1(8167):514. doi:10.1016/s0140-6736(80)92767-1 6102236

[22] Horne R , Weinman J , Hankins M . The beliefs about medicines questionnaire: the development and evaluation of a new method for assessing the cognitive representation of medication. Psychol Health. 1999;14(1):1‐24. doi:10.1080/08870449908407311

[23] Salkovskis PM , Rimes KA , Warwick HMC , Clark DM . The health anxiety inventory: development and validation of scales for the measurement of health anxiety and hypochondriasis. Psychol Med. 2002;32(5):843‐853. doi:10.1017/S0033291702005822 12171378

[24] HIQA . Guidelines for the economic evaluation of health Technologies in Ireland 2020. Published Online 2020:108. https://www.hiqa.ie/sites/default/files/2020-09/HTA-Economic-Guidelines-2020.pdf

[25] Department of Health . 1 February and 1 June 2022 Consolidated pay scales. Health Sector Consolidated Salary Scales in Accordance with the FEMPI Acts, the Public Service Agreements and the Public Service Pay and Pensions Act 2017. 2022. Accessed November 1, 2022. https://healthservice.hse.ie/staff/pay/pay-scales/

[26] “ Central Statistics Office .” Consumer Price Index ‐ CSO. https://www.cso.ie/en/statistics/prices/consumerpriceindex/

[27] Alexander SPH , Fabbro D , Kelly E , et al. THE CONCISE GUIDE TO PHARMACOLOGY 2021/22: catalytic receptors. Br J Pharmacol. 2021;178(S1):S264‐S312. doi:10.1111/bph.15541 34529829

[28] HSE Primary Care Reimbursement Service. Product Updates Notification ‐ G.M.S. Reimbursable Items. https://www.sspcrs.ie/libr/html/monthlyproductupdate.pdf

[29] Jin R , Nduka C , Courmier D , et al. Real‐world experience of adalimumab biosimilar (ABP 501) use in patients with inflammatory bowel disease in Europe. Adv Ther. 2024;41(1):331‐348. doi:10.1007/s12325-023-02712-w 37957522 PMC10796661

[30] Müller‐Ladner U , Dignass A , Gaffney K , et al. The PROPER study: a 48‐week, pan‐European, real‐world study of biosimilar SB5 following transition from reference adalimumab in patients with immune‐mediated inflammatory disease. BioDrugs. 2023;37(6):873‐889. doi:10.1007/s40259-023-00616-3 37632666 PMC10581927

[31] Bouhnik Y , Carbonnel F , Fumery M , et al. The PERFUSE study: the experience of patients receiving adalimumab biosimilar SB5. Dig Liver Dis. 2023;55(12):1658‐1666. doi:10.1016/j.dld.2023.05.025 37308394

[32] Tursi A , Mocci G , Allegretta L , et al. Comparison of performances of adalimumab biosimilars SB5, ABP501, GP2017, and MSB11022 in treating patients with inflammatory bowel diseases: a real‐life, multicenter, observational study. Inflamm Bowel Dis. 2023;29(3):376‐383. doi:10.1093/ibd/izac092 35579320

[33] Queiroz NSF , Saad‐Hossne R , Fróes RD , et al. Discontinuation rates following a switch from a reference to a biosimilar biologic in patients with inflammatory bowel disease: a systematic review and meta‐analysis. Arq Gastroenterol. 2020;57(3):232‐243. doi:10.1590/s0004-2803.202000000-45 32935741

[34] Boone NW , Liu L , Romberg‐Camps MJ , et al. The nocebo effect challenges the non‐medical infliximab switch in practice. Eur J Clin Pharmacol. 2018;74(5):655‐661. doi:10.1007/s00228-018-2418-4 29368188 PMC5893662

[35] Sarnola K , Merikoski M , Jyrkkä J , Hämeen‐Anttila K . Physicians' perceptions of the uptake of biosimilars: a systematic review. BMJ Open. 2020;10(5):e034183. doi:10.1136/bmjopen-2019-034183 PMC7228507 32371511

[36] Evers AWM , Colloca L , Blease C , et al. What should clinicians tell patients about placebo and nocebo effects? Practical considerations based on expert consensus. Psychother Psychosom. 2020;90(1):49‐56. doi:10.1159/000510738 33075796

[37] Stone JK , Shafer LA , Graff LA , et al. The association of efficacy, optimism, uncertainty and health anxiety with inflammatory bowel disease activity. J Psychosom Res. 2022;154:110719. doi:10.1016/j.jpsychores.2022.110719 35065327

[38] Glintborg B , Sørensen IJ , Loft AG , et al. A nationwide non‐medical switch from originator infliximab to biosimilar CT‐P13 in 802 patients with inflammatory arthritis: 1‐year clinical outcomes from the DANBIO registry. Ann Rheum Dis. 2017;76(8):1426‐1431. doi:10.1136/annrheumdis-2016-210742 28473425

[39] Jørgensen KK , Olsen IC , Goll GL , et al. Switching from originator infliximab to biosimilar CT‐P13 compared with maintained treatment with originator infliximab (NOR‐SWITCH): a 52‐week, randomised, double‐blind, non‐inferiority trial. Lancet. 2017;389(10086):2304‐2316. doi:10.1016/S0140-6736(17)30068-5 28502609

[40] Derikx LAAP , Dolby HW , Plevris N , et al. Effectiveness and safety of adalimumab biosimilar SB5 in inflammatory bowel disease: outcomes in originator to SB5 switch, double biosimilar switch and bio‐Naïve SB5 observational cohorts. J Crohns Colitis. 2021;15(12):2011‐2021. doi:10.1093/ecco-jcc/jjab100 34089587 PMC8684477

