# Supplementary material for: A single‐centre analysis of a biosimilar switching programme for adalimumab in inflammatory bowel disease
Source: Br J Clin Pharmacol. 2025 Apr 29;91(9):2628–35. doi: 10.1002/bcp.70086 (PMC12381624; doi:10.1002/bcp.70086)
Supplement: Supplementary file 1 — FIGURE S1 Harvey–Bradshaw Index (HBI) scores pre‐ and postswitch. FIGURE S2 Faecal calprotectin (FC; measured in μg/g) measurements pre‐ and postswitch. FIGURE S3 C‐reactive protein (CRP) measurements pre‐ and postswitch. TABLE S1 Switching clinic staff costs. TABLE S2 Unscheduled care costs (2 patients, 8‐week time horizon). TABLE S3 Mean drug cost calculation. TABLE S4 Occurrence of adverse effects. [file BCP-91-2628-s001.docx]

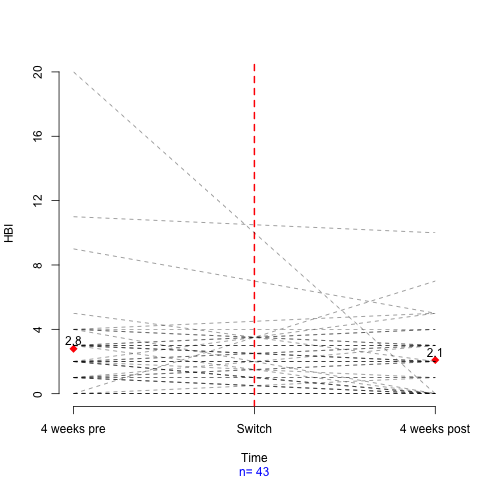


Supplemental Figure 1: Harvey-Bradshaw Index (HBI) scores pre- and post-switch


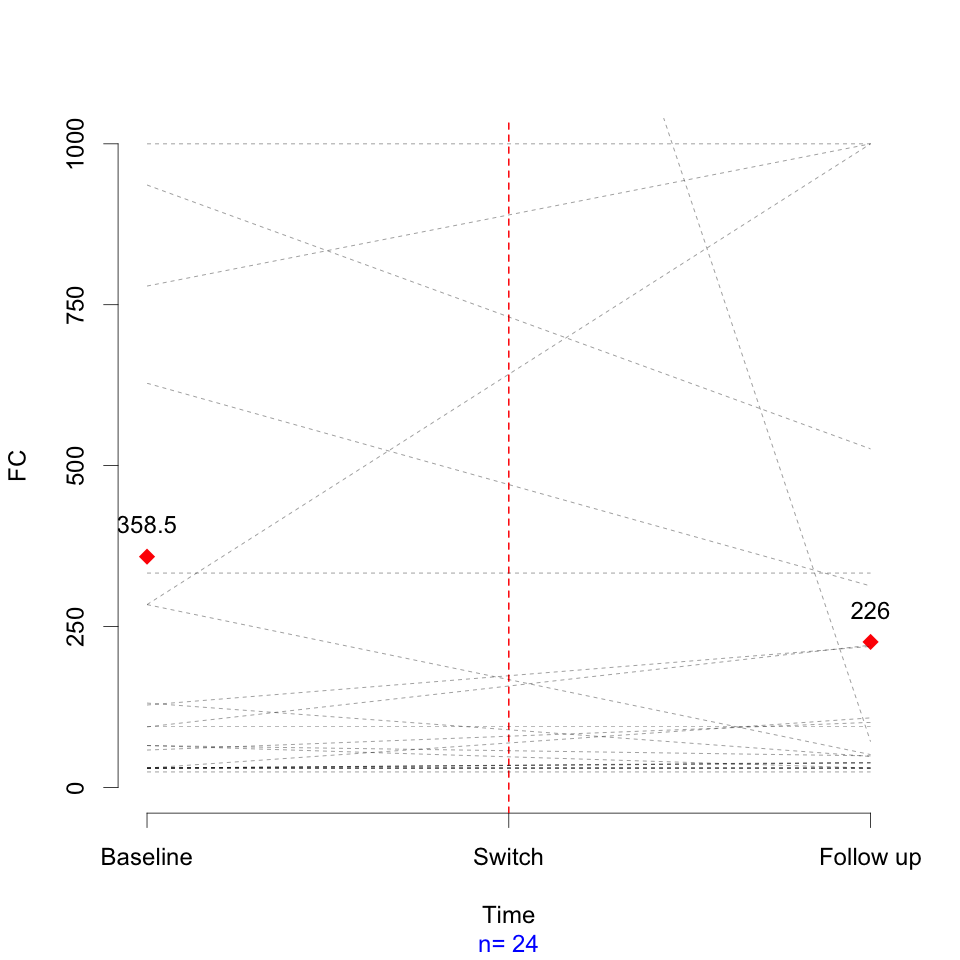


Supplemental Figure 2: Faecal Calprotectin (FC; measured in μg/g) measurements pre- and post-switch.


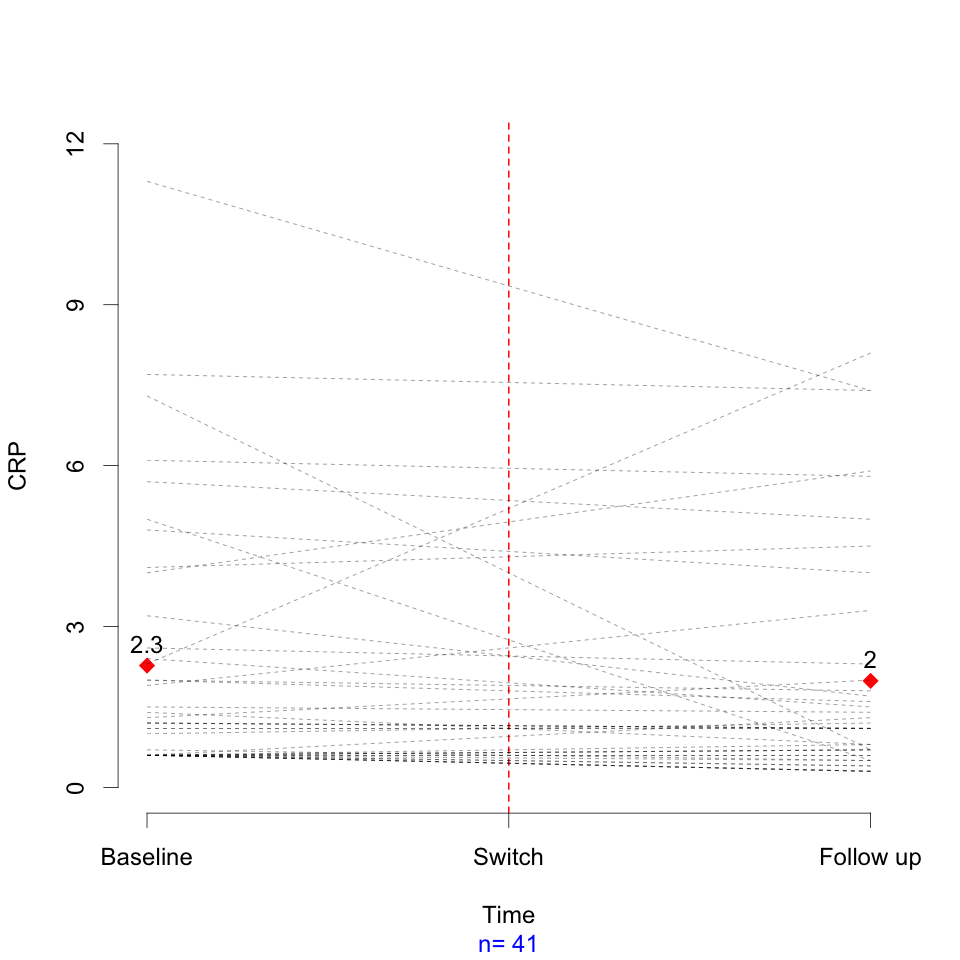
Supplemental Figure 3: C-reactive protein (CRP) measurements pre- and post-switch


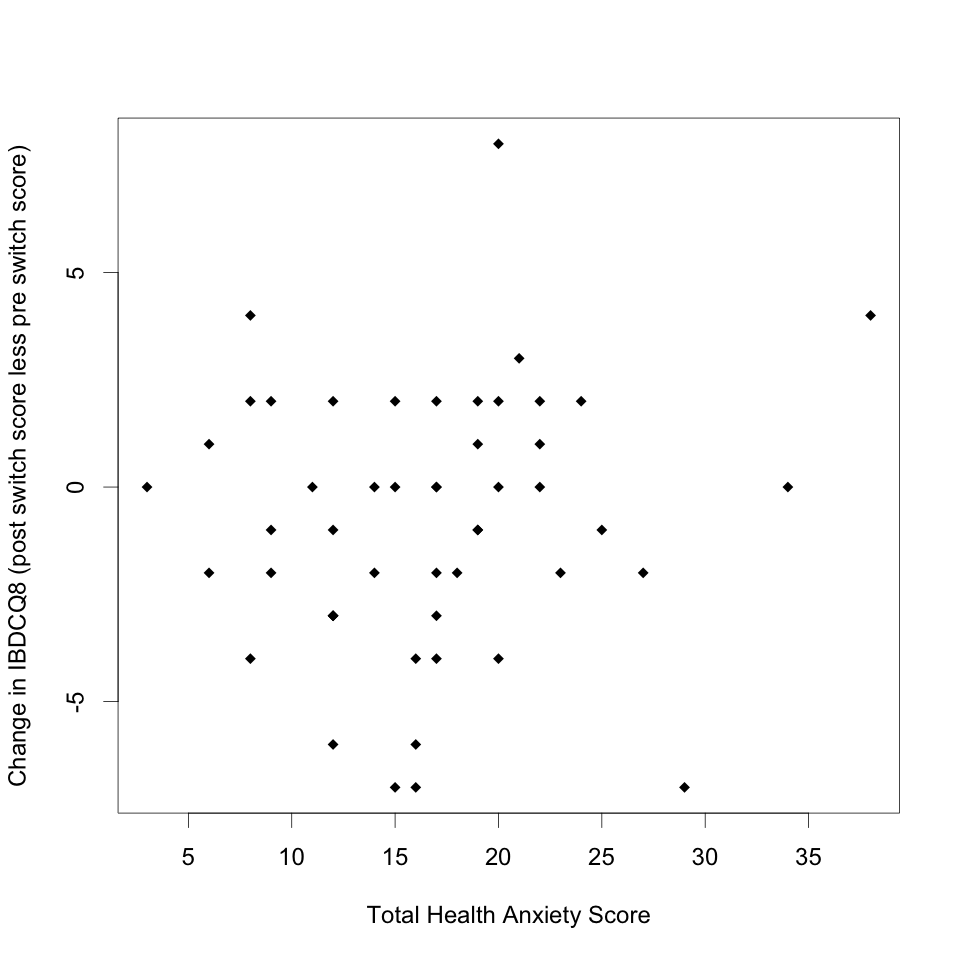


Supplemental Figure 4: Relationship between change in score on IBD control questionnaire (IBDCQ) and the score on the short form Health Anxiety Index (Total Health Anxiety Score).

Supplementary Table 1 – Switching clinic staff costs.

| ​ | Hourly rate (€)​ | Number of hours​ | Total (€)​ |
| --- | --- | --- | --- |
| Staff nurse administrative time​ | 37.36​ | 36​ | 1345.11​ |
| ANP administrative time​ | 60.80​ | 5​ | 304.00​ |
| Staff nurse time in clinic​ | 37.36​ | 41.75​ | 1559.95​ |
| ANP time in clinic​ | 60.80​ | 53.75​ | 3268.00​ |
| SpR -admin and production of PIL​ | 65.34​ | 20​ | 1306.80​ |
| Consultant supervision​ | 167.97​ | 10​ | 1679.72​ |
| ​ | ​ | ​ | ​ |
| Total staff costs​ | ​ | ​ | **9463.56**​ |

Supplementary Table 2 – Unscheduled care costs (2 patients, 8-week time horizon)

|  | Patient 1​ | Patient 2​ | Total Units​ | Unit cost corrected to 2021  ​ | Total​ | Source for unit cost |
| --- | --- | --- | --- | --- | --- | --- |
| Nurse phone call (min)​ | 30.40​ | 10.13​ | 40.53 | € 1.01 ​ | € 41.07 ​ | https://assets.hse.ie/media/documents/October_2021_consolidated_salary_scales.pdf |
| OPD visit (N)​ | 2​ | 1​ | 3​ | € 176.27 ​ | € 528.82 ​ | HPO - https://www.hpo.ie/seminar/pdf/2022/Pilot-OP-ABFConference2022.pdf |
| GP visit​ | 0​ | 1​ | 1​ | € 52.17 ​ | € 52.17 ​ | Smith S, Jiang J, Normand C and O’Neill C. Unit costs for non-acute care in Ireland 2016—2019. HRB Open Res 2021, 4:39 (https://doi.org/10.12688/hrbopenres.13256.1) |
| Ambulance transfer​ | 0​ | 1​ | 1​ | € 705.53 ​ | € 705.53 ​ | https://www.hiqa.ie/sites/default/files/2017-02/Mechanical-Thrombectomy-technical-report.pdf |
| AMU visit​ | 0​ | 1​ | 1​ | € 294.52 ​ | € 294.52 ​ | Healthcare Pricing Office, HSE (taken to be equal to an ED visit) |
| Total​ | ​ | ​ | ​ | ​ | € 1,622.11 ​ |  |
| Mean per patient ​  (=total unscheduled care costs /64)​ | ​ | ​ | ​ | ​ | € 25.35 ​ |  |

Supplementary Table 3 – Mean drug cost calculation

| ​ | N​ | Monthly Cost per patient​ | Total Monthly Cost​ |
| --- | --- | --- | --- |
| Humira 40mg q 2/52​ | 44​ | 2,209.44​ | 97,215.36​ |
| Humira 40mg q 1/52​ | 19​ | 4,418.88​ | 83,958.72​ |
| Humira 80mg q 2/52​ | 1​ | 2,159.90​ | 2,159.90​ |
| Total​ | 64​ | ​ | 183,333.98​ |
| Humira mean cost per patient per month​ | | | 2,864.59​ |
| ​ | ​ | ​ | ​ |
| Amgevita 40mg q 2/52​ | 44​ | 1,325.66​ | 58,329.04​ |
| Amgevita 40mg q 1/52​ | 19​ | 2,651.32​ | 50,375.08​ |
| Amgevita 80mg q 2/52​ | 1​ | 2,651.32​ | 2,651.32​ |
| ​ | 64​ | ​ | 111,355.44​ |
| Amgevita mean cost per patient per month​ | | | 1,739.93​ |

Supplementary Table 4 – Occurrence of adverse effects

|  | **Injection site reactions** | **Other ADRs** |
| --- | --- | --- |
| Pre-switch | 0 | 0 |
| Post-switch | 6 | 11* |
| (McNemar’s test) | *alpha*=0.05, *p* = 0.03125. | *alpha=0.05* with *p* = 9*.*8×10^−4^. |
| * 4 Headaches, 2 Malaise, 2 Joint pain, 2 Nausea, 1 Low back pain, 1 Fatigue, 1 Rash | | |
